# CAR T cell therapy for breast cancer: harnessing the tumor milieu to drive T cell activation

**DOI:** 10.1186/s40425-018-0347-5

**Published:** 2018-05-10

**Authors:** Pradip Bajgain, Supannikar Tawinwung, Lindsey D’Elia, Sujita Sukumaran, Norihiro Watanabe, Valentina Hoyos, Premal Lulla, Malcolm K. Brenner, Ann M. Leen, Juan F. Vera

**Affiliations:** 1 Center for Cell and Gene Therapy, Baylor College of Medicine, Texas Children’s Hospital and Houston Methodist Hospital, Houston, TX 77030 USA; 2 Interdepartmental Program in Translational Biology and Molecular Medicine, Baylor College of Medicine, Houston, TX 77030 USA; 3 Department of Pharmacology and Physiology, Faculty of Pharmaceutical Sciences, Chulalongkorn University, Bangkok, 10330 Thailand

**Keywords:** Chimeric antigen receptor, Genetic engineering, Inverted cytokine receptor, T cell therapy, Breast cancer

## Abstract

**Background:**

The adoptive transfer of T cells redirected to tumor via chimeric antigen receptors (CARs) has produced clinical benefits for the treatment of hematologic diseases. To extend this approach to breast cancer, we generated CAR T cells directed against mucin1 (MUC1), an aberrantly glycosylated neoantigen that is overexpressed by malignant cells and whose expression has been correlated with poor prognosis. Furthermore, to protect our tumor-targeted cells from the elevated levels of immune-inhibitory cytokines present in the tumor milieu, we co-expressed an inverted cytokine receptor linking the IL4 receptor exodomain with the IL7 receptor endodomain (4/7ICR) in order to transform the suppressive IL4 signal into one that would enhance the anti-tumor effects of our CAR T cells at the tumor site.

**Methods:**

First (1G - CD3ζ) and second generation (2G - 41BB.CD3ζ) MUC1-specific CARs were constructed using the HMFG2 scFv. Following retroviral transduction transgenic expression of the CAR±ICR was assessed by flow cytometry. In vitro CAR/ICR T cell function was measured by assessing cell proliferation and short- and long-term cytotoxic activity using MUC1+ MDA MB 468 cells as targets. In vivo anti-tumor activity was assessed using IL4-producing MDA MB 468 tumor-bearing mice using calipers to assess tumor volume and bioluminescence imaging to track T cells.

**Results:**

In the IL4-rich tumor milieu, 1G CAR.MUC1 T cells failed to expand or kill MUC1+ tumors and while co-expression of the 4/7ICR promoted T cell expansion, in the absence of co-stimulatory signals the outgrowing cells exhibited an exhausted phenotype characterized by PD-1 and TIM3 upregulation and failed to control tumor growth. However, by co-expressing 2G CAR.MUC1 (signal 1 - activation + signal 2 - co-stimulation) and 4/7ICR (signal 3 - cytokine), transgenic T cells selectively expanded at the tumor site and produced potent and durable tumor control in vitro and in vivo.

**Conclusions:**

Our findings demonstrate the feasibility of targeting breast cancer using transgenic T cells equipped to thrive in the suppressive tumor milieu and highlight the importance of providing transgenic T cells with signals that recapitulate physiologic TCR signaling – [activation (signal 1), co-stimulation (signal 2) and cytokine support (signal 3)] - to promote in vivo persistence and memory formation.

**Electronic supplementary material:**

The online version of this article (10.1186/s40425-018-0347-5) contains supplementary material, which is available to authorized users.

## Background

Breast cancer is the most prevalent malignant disease of women in the developed world and remains one of the leading causes of death; in 2017 an estimated 252,710 new cases of invasive breast cancer were diagnosed in women [1]. Although early detection and advances in conventional chemo-, radio-, and antibody-based therapies have substantially increased cure rates (99% 5-year survival in patients with localized disease), the 5-year survival of those with distant metastases is only 27%, highlighting the need for novel therapies [1].

The adoptive transfer of T cells modified to express tumor-targeted chimeric antigen receptors (CARs) has proven to be effective for the treatment of a range of refractory hematologic malignancies including ALL, B-CLL, and lymphoma and holds promise for the treatment of solid tumors [2–6]. However, extension of this approach to metastatic breast cancer requires both the identification of an appropriate antigen to target and consideration of additional genetic strategies to protect these cells from the suppressive tumor microenvironment (TME). Indeed, the breast cancer TME is infiltrated by regulatory T cells [7, 8], myeloid-derived suppressor cells (MDSCs) [9, 10], and rich in inhibitory/Th2-polarized cytokines such as IL4 [11–13], that promote tumor survival [14–17], migration and invasion [18, 19], and directly inhibit Th1-polarized effector T cells [20, 21].

We now explore the feasibility of targeting metastatic breast cancer using T cells modified with a CAR targeting the tumor associated antigen (TAA) mucin1 (MUC1), whose overexpression in underglycosylated form has been associated with tumor invasiveness and metastatic potential [22–28]. Further, to ensure that our CAR T cells remain operative in the tumor microenvironment, we co-express an inverted cytokine receptor (ICR) encoding the cytokine-binding portion of the IL4 receptor exodomain linked to the immunostimulatory IL7 receptor signaling endodomain (4/7ICR) [29, 30]. We demonstrate the potent, selective, and sustained anti-tumor activity of these dual transgenic T cells in the IL4-rich breast cancer microenvironment and highlight the importance of transgenically delivering a combination of signals that recapitulate physiological T cell signaling (activation, co-stimulation, and cytokine support) to ensure durable benefit.

## Methods

### Donor and cell lines

Peripheral blood mononuclear cells (PBMCs) were obtained from healthy volunteers after informed consent on protocols approved by the Baylor College of Medicine Institutional Review Board. The cell lines MDA MB 468, MCF-7, and 293T were obtained from the American Type Culture Collection (Rockville, MD) and were grown in Dulbecco’s Modified Eagle Medium (DMEM, GE Healthcare Life Sciences, Pittsburgh, PA) supplemented with 10% heat-inactivated fetal bovine serum (FBS) (HyClone, Waltham, MA) and 2 mM L-GlutaMAX (Gibco BRL Life Technologies, Inc., Gaithersburg, MD). All cell lines were maintained in a humidified atmosphere containing 5% carbon dioxide (CO_2_) at 37 °C.

### Generation of retroviral constructs and retroviral supernatant

We synthesized a human, codon-optimized 1st generation CAR [31] with specificity against tumor-associated MUC1 using the published HMFG2 scFv sequence [32–34], which was cloned in-frame with the IgG2-CH3 domain (spacer) and the zeta (ζ) chain of the T cell receptor (TCR) CD3 complex in an SFG retroviral backbone to make the 1st generation CAR (1G). To generate the 2G.CAR, the 41BB co-stimulatory endodomain was added to the 1G construct between the CD28 transmembrane and ζ domains.

To generate the 4/7ICR, we synthesized (DNA 2.0, Menlo Park, CA) a codon-optimized sequence encoding the signal peptide and extracellular domain of the human IL4 receptor α chain fused with the transmembrane and intracellular domain of IL7 receptor, with the restriction sites *Xho*1 and *Mlu*1 incorporated up and downstream, respectively [29, 30]. The 4/7ICR DNA insert was incorporated into an SFG retroviral vector that contained the fluorescent marker mOrange. Retroviral supernatant for both the CARs and 4/7ICR was generated as previously described [29].

### Generation of CAR T cells

To generate CAR T cells, 1 × 10^6^ PBMCs were plated in each well of a non-tissue culture-treated 24-well plate that had been pre-coated with OKT3 (1 mg/ml) (Ortho Biotech, Inc., Bridgewater, NJ) and CD28 (1 mg/ml) (Becton Dickinson & Co., Mountain View, CA). Cells were cultured in complete media [RPMI-1640 containing 45% Clicks medium (Irvine Scientific, Inc., Santa Ana, CA), 10% FBS, and 2 mM L-GlutaMAX], which was supplemented with recombinant human IL2 (50 U/mL, NIH, Bethesda, MD) on day 1. On day 3 post OKT3/CD28 T blast generation, 1 mL of retroviral supernatant was added to a non-tissue culture-treated 24-well plate pre-coated with recombinant fibronectin fragment (FN CH-296; Retronectin; Takara Shuzo, Otsu, Japan) and centrifuged at 2000G for 90 min. OKT3/CD28 activated T cells (0.2 × 10^6^/mL) were resuspended in complete media supplemented with IL2 (100 U/mL) and then added to the wells and centrifuged at 400G for 5 min. To generate CAR and 4/7ICR co-expressing cells, activated T cells were transduced sequentially first with either 1G or 2G CAR.MUC1 and then with 4/7ICR on days 3 and 4, respectively. Transduction efficiency was measured 3 days post transduction by flow cytometry.

### MDA MB 468 transduction

We generated an MDA MB 468 cell line that expressed transgenic MUC1 and produced IL4 to ensure homogeneous expression of these molecules. To do this, IL4 cytokine-mOrange retroviral supernatant was plated in a non-tissue culture-treated 24-well plate (1 ml/well), which was pre-coated with a recombinant fibronectin fragment. MDA MB 468 cells (0.2 × 10^6^/mL) were added to the plates (1 mL/well) and then transferred to a 37°C, 5% CO_2_ incubator. Transgene expression was analyzed by flow 1-week post-transduction and was confirmed by IL4 ELISA (R&D Systems, Minneapolis, MN), performed per manufacturer instructions. After 2 weeks, these cells were further transduced with a retroviral vector encoding MUC1 [35]. A truncated CD19 (dCD19) [36] was incorporated into the MUC1 vector using an internal ribosome entry site element to facilitate transgene detection. Cells were subsequently sorted based on mOrange and dCD19 expression using a MoFlo flow cytometer (Cytomation, Fort Collins, CO).

### Flow cytometry

The following antibodies were used in this study for T cell phenotyping: CD3-PerCP (clone SK7/Cat# 347344), CD25-APC AF700, CD4-Krome Orange (13B8.2/A96417), CD8-Pacific Blue (B9.11/A82791), CD3-APC (Beckman Coulter Inc. Brea, CA), Rat Anti-Mouse IgG1-APC (X56/550874) (BD Biosciences, San Jose, CA). PD-1-Percp Cy7 and TIM3-APC (BD Biosciences, San Jose, CA) were used as markers of T cell exhaustion. MUC1 antigen expression by tumor cells was measured using anti-MUC1, (Santa Cruz Biotechnology. Inc., Dallas, TX). CAR molecules were detected using Goat anti-human F(ab’)2 antibody conjugated with AlexaFluor647 (109–606-097) (Jackson ImmunoResearch Laboratories, Inc., West Grove, PA). Cells were stained with saturating amounts of antibody (~5uL) for 20 min at 4 °C, washed (PBS, Sigma-Alrich, St. Louis, MO), and then acquired on Gallios™ Flow Cytometer (Beckman Coulter Inc., Brea, CA). Analysis was performed using Kaluza® Flow Analysis Software (Beckman Coulter Inc.).

### ^51^Chromium-release assay

The cytotoxicity and specificity of engineered T cells was evaluated in a standard 4–6 hr ^51^Cr-release assay, as previously described [37].

### T cell stimulation assay

To measure T cell expansion upon antigen stimulation in the presence of IL4 cytokine (400 pg/mL) (R&D Systems, Minneapolis, MN), 1 × 10^6^ CAR.MUC1 T cells were cultured with 0.5 × 10^6^ irradiated MDA MB 468 tumor cells engineered to overexpress MUC1. Tumor cells were irradiated (100Gy) to halt their expansion using Rad Source RS2000 Biological X-Ray Irradiator (Rad Source Technologies, Buford, GA). IL4 was added to culture 2 times per week and T cells were quantified by trypan blue exclusion.

### Co-culture experiments

For co-culture experiments, eGFP-FFLuc+ MDA MB 468 overexpressing MUC1 (1 × 10^6^ cells) were inoculated into 3D algimatrix bioscaffold (Thermo Fisher Scientific, Inc., Waltham, MA) and cultured in 6-well G-Rex [38] devices (Wilson Wolf Manufacturing, New Brighton, MN). Three days later, CAR or CAR+ICR-modified T cells were added to tumor cells to achieve a T cell:tumor cell ratio of 1:10 in the presence of 400 U/mL IL4. Anti-tumor activity was monitored using the IVIS Lumina In Vivo Imaging system (Caliper Life Sciences, Hopkinton, MA) 10 min after adding D-luciferin (PerkinElmer, Waltham, MA) (15 mg/mL) into the culture media. Cells were then collected and recovered from the Algimatrix using Aligmatrix dissolving buffer (Thermo Fisher Scientific, Inc.). To quantify cells by flow cytometry, we used CountBright™ Absolute Counting Beads (C36950; Invitrogen, Eugene, OR) and 7-AAD was added to exclude dead cells. Acquisition was halted at 5000 beads. T cells were then purified using CD3 microbead column (MACS) for subsequent ^51^Cr-release assay.

### In vivo study

Six to eight-week-old female NSG mice (NOD.Cg-Prkdcscid Il2rgtm1Wjl/SzJ, Jackson ImmunoResearch Laboratories, Inc., West Grove, PA) were injected with 5 × 10^6^ IL4 cytokine-producing, MUC1-overexpressing MDA MB 468 (MDA MB 468/IL4) cells suspended in 50%DPBS/50%matrigel subcutaneously (s.c.) into the left inferior mammary fat pad. Once the tumor reached a size of approx. 75 mm^3^ (~ 4–5 weeks), animals were injected intravenously (i.v.) with 3 × 10^6^ eGFP-FFLuc+1G, 1G.4/7ICR, 2G, or 2G.4/7ICR T cells. Tumor size was measured by bi-weekly caliper measurement and tumor volume (mm^3^) was calculated by length x width x width/2. T cell expansion and persistence was monitored using the IVIS Lumina In Vivo Imaging system (Caliper Life Sciences, Hopkinton, MA) 10 min after injection (i.p.) with 100ul of D-luciferin (15 mg/mL). All in vivo analysis was performed using Living Image software (Caliper Life Sciences, Inc., Hopkinton, MA). Experiments were performed according to Baylor College of Animal Husbandry guidelines.

### Statistical analysis

Results are reported as mean ± SEM unless stated otherwise. All statistical analyses were performed using GraphPad Prism software. Statistical significance between/among groups were determined using one-way ANOVA, two-way ANOVA, or unpaired two-tailed t tests. *P*-values less than 0.05 were considered statistically significant.

## Results

### 4/7ICR improves the cytolytic function and proliferation of CAR.MUC1 T cells in presence of IL4

**Fig. 1:**
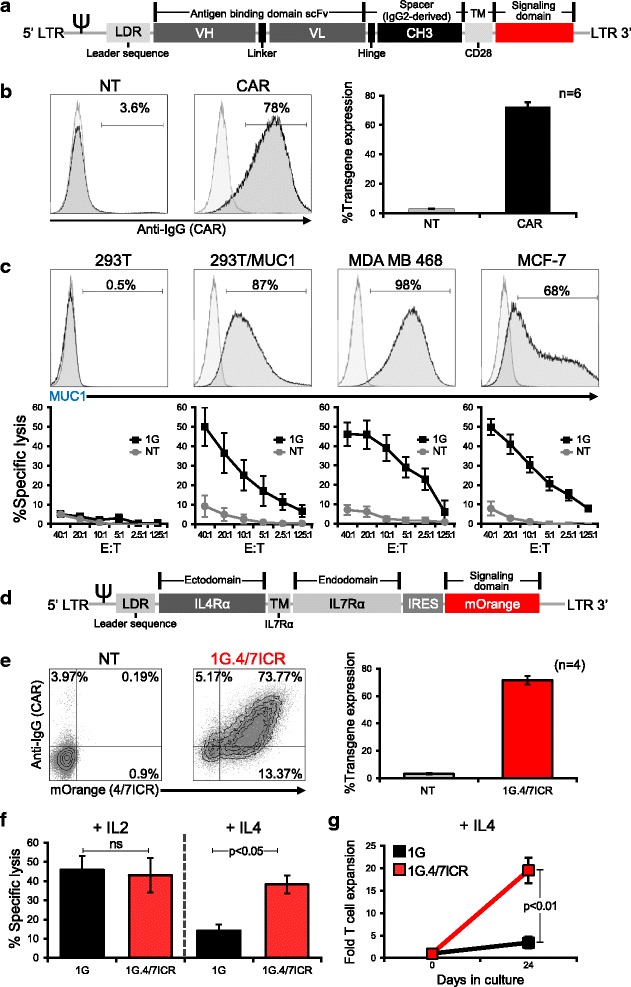
4/7ICR improves the cytolytic function and proliferation of CAR.MUC1 T cells in presence of IL4. **a** Schematic representation of 1st generation CAR.MUC1 (1G) construct. **b** CAR.MUC1 expression on activated T cells measured 3 days post-transduction (representative donor on the left, summary data on the right). Data represents mean ± SEM (*n* = 6). **c** Phenotypic analysis of MUC1 expression on different cell lines (top panel) and in vitro cytolytic function of control (NT) and CAR T cells assessed in a 5 hr ^51^Cr-release assay at E:Ts of 1.25:1 to 40:1, using MUC1+ targets (293T/MUC1, MDA MB 468, MCF-7) and MUC1- target (293T) (bottom). Data represents mean ± SEM (*n* = 5). **d** Schematic of 4/7ICR vector map. **e** Transgenic expression of both 4/7ICR and CAR.MUC1 in T cells as detected by mOrange and anti-IgG, respectively. Right panel shows summary data representing the percentage of double-positive cells (1G.4/7ICR) (mean ± SEM, *n* = 4). **f** Cytolytic function of transgenic (1G or 1G.4/7ICR) T cells pre-exposed to IL4 as assessed in a 4 hr ^51^Cr-release assay using MDA MB 468 as a target at the indicated E:T ratios. Statistical significance was calculated between 1G and 1G.4/7ICR using One-way ANOVA, *p* < 0.05. **g** Cell expansion of 1G or 1G.4/7ICR T cells (1 × 10^6^) stimulated weekly with irradiated MDA MB 468 cells (0.5 × 10^6^) with IL4 (400 U/mL) added twice weekly. T cell expansion was quantified by cell counting using trypan blue exclusion to assess cell viability. Statistical significance was calculated between 1G and 1G.4/7ICR using One-way ANOVA, *p* < 0.01

To target breast cancer, we generated a retroviral vector encoding a first-generation human, codon-optimized CAR (1G) directed against the tumor-associated antigen MUC1 (Fig. 1a) [35]. This transgenic molecule could be stably expressed on activated T cells (mean 72.3 ± 1.9% transduction efficiency, Fig. 1b), enabling CAR T cells to specifically kill MUC1-expressing tumors (293T/MUC1, MDA MB 468, and MCF-7) with no recognition of MUC1 negative targets (293T) (Fig. 1c). In the breast cancer tumor microenvironment, antigen-specific T cells can be rendered dysfunctional following chronic exposure to immunosuppressive cytokines. These include IL4, which is elevated in patients with breast cancer [11–13]. To ensure that our CAR.MUC1 T cells persist and remain functional at the tumor site, we developed an inverted chimeric cytokine receptor “4/7ICR” (Fig. 1d) containing the cytokine-binding portion of the IL4 receptor linked with the signaling endodomain of IL7 receptor, which we co-expressed with 1G CAR [Fig. 1e -mean 71.5 ± 3% double positive (1G.4/7ICR) T cells]. To test if the 4/7ICR enabled CAR T cells to withstand the inhibitory effects of IL4, we cultured 1G or 1G.4/7ICR T cells with either IL2 or IL4 for 14 days and subsequently assessed their cytolytic function in a 4 hr ^51^Cr-release assay using MDA MB 468 cells as targets. As shown in Fig. 1f, there was no difference between the cytolytic potential of 1G or 1G.4/7ICR T cells cultured with IL2. However, when exposed to IL4, the cytolytic capacity of unprotected 1G T cells was significantly less than that of 1G.4/7ICR T cells (14.2 ± 3.2% vs 38.3 ± 4.8% specific lysis, E:T ratio 20:1; *p* < 0.05). Similarly, the expansion of 1G.4/7ICR T cells was superior to their 1G counterparts when we cultured both in the presence of recombinant IL4 with weekly antigen stimulation from irradiated MDA MB 468 tumor cells (Fig. 1g).

### Transgenic expression of 4/7ICR is insufficient to overcome tumor-mediated T cell dysfunction

**Fig. 2:**
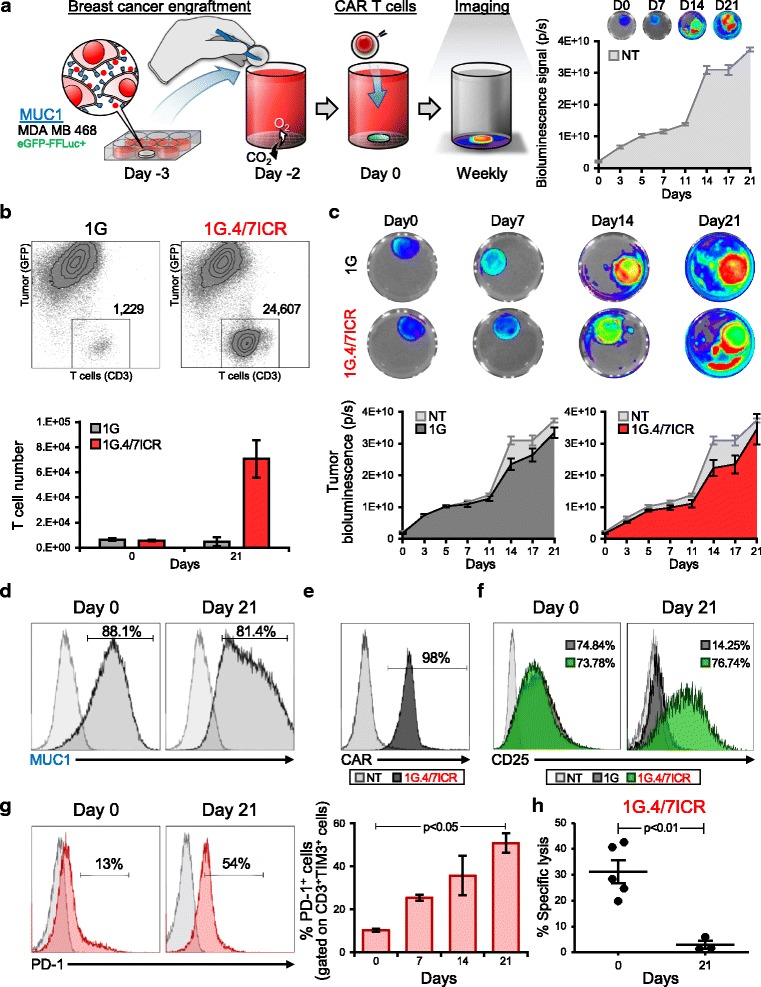
Transgenic expression of 4/7ICR is insufficient to overcome tumor-mediated T cell dysfunction. **a** Schematic of co-culture experimental setup (left panel) and bioluminescence data tracking tumor expansion (in the absence of T cell treatment) over time (right panel). **b** Representative dot plots (top) and summary quantitative data (bottom) showing T cells and tumor cell numbers on day 0 and day 21 of coculture (mean ± SEM, n = 6 independent experiments). Significance was determined by an unpaired two-tailed t-test, p < 0.05, 1G.4/7ICR compared with 1G. **c** Representative images of bioluminescent tumor cells (top) and summarized quantitative bioluminescence signal from tumor cells treated with either NT, 1G or 1G.4/7ICR T cells over time (mean ± SEM, n = 6). **d** Phenotypic analysis of MUC1 expression on MDA MB 468 cells on day 0 and day 21 of co-culture (representative donor). **e** CAR expression on 1G.4/7ICR cells after co-culture with tumor cells. **f** CD25 expression on 1G versus 1G.4/7ICR T cells on day 0 and day 21 of co-culture (light grey: isotype control, dark grey: 1G T cells, green: 1G.4/7ICR T cells). **g** Surface expression of PD-1 (representative donor - left, summary data - right) on 1G.4/7ICR cells gated on CD3^+^TIM3^+^cells and analyzed on days 0, 7, 14 and 21 of co-culture (mean ± SEM, n = 6, p < 0.05). **h** Cytolytic activity of 1G.4/7/ICR prior to and day 21 post-coculture using MDA MB 468 cells as targets (E:T 10:1; mean ± SEM, n = 4, p < 0.01)

We next explored whether co-expression of the 4/7ICR and the 1G CAR produced superior anti-tumor effects in a long-term tumor model that recapitulated an IL4-rich milieu. We co-cultured GFP-firefly luciferase (eGFP-FFLuc) labeled MDA MB 468 breast cancer cells with either 1G or 1G.4/7ICR T cells at an effector-to-target (E:T) ratio of 1:10 in the presence of IL4 (400 U/mL), monitoring anti-tumor activity by bioluminescence imaging (Fig. 2a). When 1G.4/7ICR T cells were exposed to tumor milieu conditions, they expanded in vitro during the 3-week co-culture (Fig. 2b), but unexpectedly failed to produce superior anti-tumor activity (Fig. 2c). To explore the mechanism of failure, we examined both the tumor and 1G.4/7ICR T cells before and after treatment and saw no change in either MUC1 antigen expression on malignant cells (Fig. 2d) or CAR expression by the T cells (Fig. 2e). Additionally, we confirmed that 1G.4/7ICR cells (unlike their 1G counterparts) retained an activated (CD25+) phenotype (Fig. 2f), confirming lack of inhibition by prolonged IL4 exposure. However, we observed a progressive increase in PD-1 and TIM3 expression over time (Fig. 2g), which inversely correlated with cytolytic function of T cells extracted on day 21 of the co-culture, as shown in Fig. 2h (day 0 vs day 21). Taken together, these data show that transgenic expression of the 4/7ICR was insufficient to protect CAR.MUC1 T cells from tumor-mediated dysfunction.

### Combining 4/7ICR with a 2G CAR preserves T cell function even under suppressive conditions

**Fig. 3:**
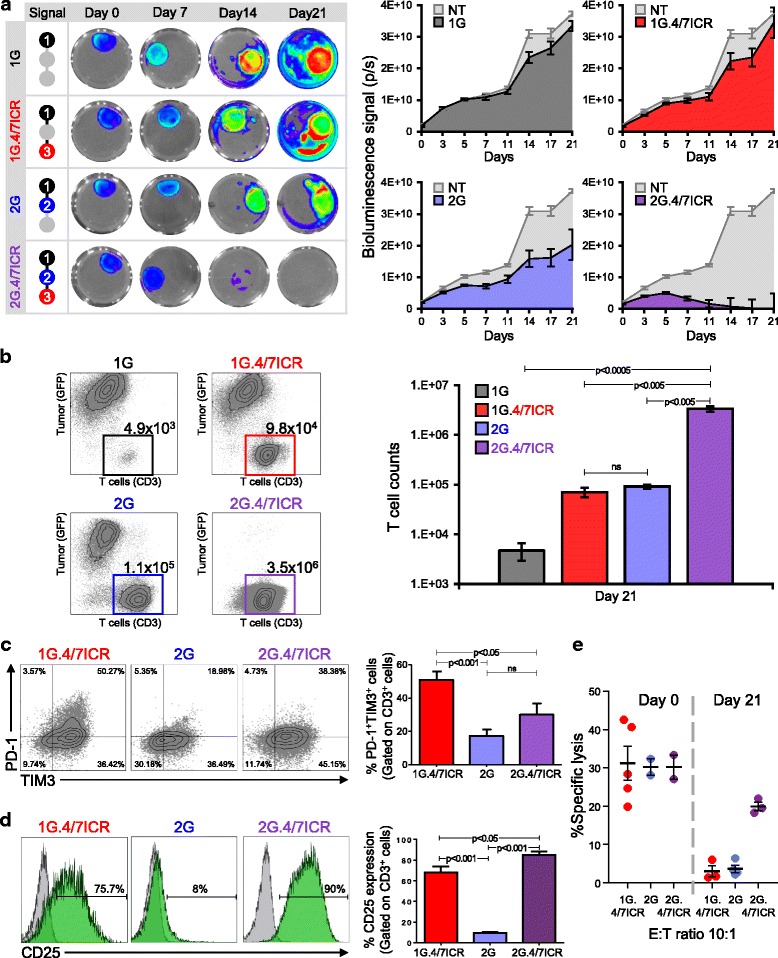
Combining 4/7ICR with a 2G CAR preserves T cell function even under suppressive conditions. **a** Serial bioluminescence imaging of eGFP-FFLuc^+^ MDA MB 468 cells co-cultured with 1G, 1G.4/7ICR, 2G, or 2G.4/7ICR T cells in the presence of IL4 [representative images - left, quantitative data - right (n = 6)]. **b** Representative dot plots and summary FACS data quantifying tumor cells and T cells after 21 days of co-culture. **c** Surface expression of PD-1 and TIM3 on transgenic T cells analyzed on day 21 after co-culture (representative data - left, summary data - right). **d** CD25 expression on 1G.4/7ICR, 2G, and 2G.4/7ICR cells on day 21 of coculture. **e** In vitro cytolytic function of 1G.4/7ICR, 2G, and 2G.4/7ICR cells isolated on day 21 after co-culture (mean ± SEM, n = 4–6). Significance was determined by two-way ANOVA. p < 0.05, p < 0.01, *p* < 0.001

T cells require 3 signals (antigen – signal 1; co-stimulation – signal 2; cytokine – signal 3) for optimal effector function and persistence in an activated state [39–41]. To determine whether the T cell exhaustion we detected in our 1G.4/7ICR cells could be overcome by incorporating a co-stimulatory signal, we constructed a 2nd generation CAR.MUC1 (2G) which contained both the CD3 zeta chain (signal 1) and a 41BB endodomain (signal 2) and could be efficiently co-expressed with the 4/7ICR (Additional file 1: Figure S1). We then compared the phenotype and function of 1G (signal 1 only), 1G.4/7ICR (signals 1 + 3), 2G (signal 1 + 2), and 2G.4/7ICR (signals 1 + 2 + 3) T cells when co-cultured with MDA MB 468 breast cancer cells in the presence of IL4 (400 U/mL) (Fig. 3). Consistent with previous observations, 1G T cells failed to expand and control tumors; while 1G.4/7ICR expanded, they were similarly unable to mediate anti-tumor effects (Fig. 3a and b). Combining signals 1 and 2 also did not produce superior T cell anti-tumor effects (Fig. 3a and b). In contrast, co-expression of 2G CAR and the 4/7ICR, which provides all 3 signals required for physiologic T cell activation and persistence, resulted in potent T cell expansion and anti-tumor activity (Fig. 3a and b), leading to durable control. Assessment of T cell phenotype post-co-culture (day 21) demonstrated a decreased expression of PD-1 and TIM3 by 2G.4/7ICR cells compared to 1G.4/7ICR T cells (Fig. 3c), and an increased expression of the activation marker CD25 (Fig. 3d). This phenotype of 2G.4/7ICR T cells correlated with their ability to kill (specific lysis of 19.9 ± 1.4%; E:T ratio of 10:1, *n* = 4, Fig. 3e) tumor cells even after a long-term (21 days) exposure to the tumor cells, while the 1G.4/7ICR and 2G cells exhibited diminished cytolytic activity (3.0 ± 1.6% and 3.7 ± 0.9% specific lysis respectively; E:T ratio of 10:1, n = 4, Fig. 3e).

### Combined expression of 4/7ICR and 2G CAR augments anti-tumor activity in vivo

**Fig. 4:**
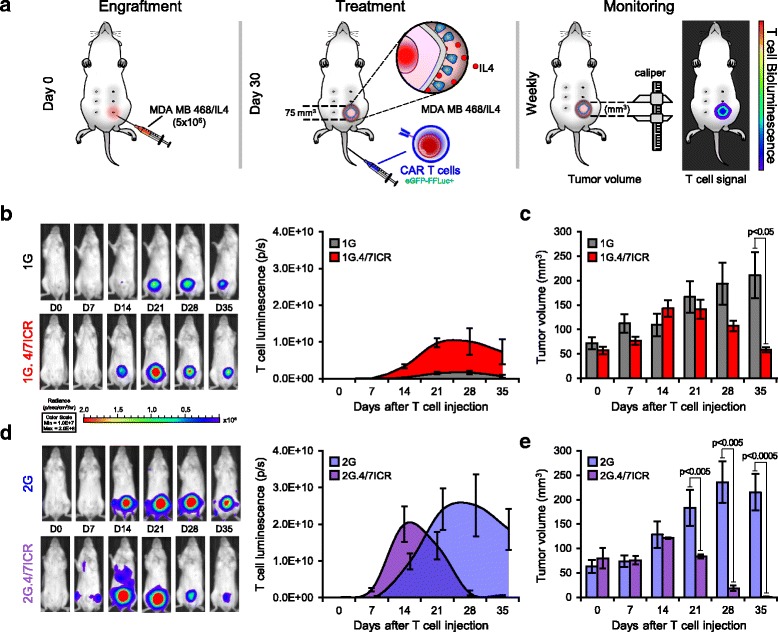
Combined expression of 4/7ICR and 2G CAR augments anti-tumor activity in vivo. **a** Schematic of in vivo experiment where NSG mice with IL4-producing MDA MB 468 cells and treated i.v. with eGFP-FFLuc+1G, 1G.4/7ICR, 2G, or 2G.4/7ICR T cells. **b** Representative animal images (left) and summary bioluminescence data (right, mean ± SEM, *n* = 3–5/group) indicating T cell localization and expansion. **c** Tumor volume measured by calipers (mean ± SEM, n = 3–5/group). Significance was determined by two-way ANOVA. p < 0.05 on day 35. **d** Representative animal images (left) and summary bioluminescence data (right, mean ± SEM, n = 3–5/group). **e** Tumor volume measured by calipers. Significance was determined by two-way ANOVA

To determine whether the potent anti-tumor effects observed when combining the 2G CAR with 4/7ICR would be recapitulated in vivo, NSG mice were engrafted (s.c. in the left inferior mammary fat pad) with 5 × 10^6^ IL4-producing MDA MB 468 cells (MDA MB 468/IL4) (Fig. 4a). Once the tumor had reached approx. 75 mm^3^ (~ 4–5 weeks post-engraftment), animals were treated with 3 × 10^6^ eGFP-FFLuc+1G, 1G.4/7ICR, 2G, or 2G.4/7ICR T cells and tracked in vivo by bioluminescence imaging. As shown in Fig. 4b and d, T cells localized at the tumor site in every group (left panel - individual examples; right panel – summary data). However, in mice treated with 1G, the T cells failed to expand (change in bioluminescence from 2.6 ± 0.35E + 07 photons/sec, day 0 to 1.69 ± 0.51E + 09 photons/sec, day 28) and the tumor rapidly outgrew (Fig. 4c). Similarly, in the 2G-treated group the tumor outgrew despite T cell expansion (Fig. 4e). In contrast, within 5 weeks of treatment, we observed a reduction in tumor in both cohorts receiving 4/7ICR-modified T cells (Fig. 4c and e). However, while none of the 1G.4/7ICR-treated animals were tumor-free, every mouse receiving 2G.4/7ICR T cells was tumor-free and remained so for an additional 4 weeks. Importantly, upon tumor clearance, the numbers of 2G.4/7ICR T cells rapidly declined (decrease in T cell signal from 2.0 ± 0.48E + 10 to 6.21 ± 2.1E + 08 photons/sec between days 14 and 35; Fig. 4d), indicating that sustained expansion required both antigen and cytokine, and supporting the safety of the approach.

### 2G.4/7ICR T cells persist long term and retain their anti-tumor activity and tumor selectivity

**Fig. 5:**
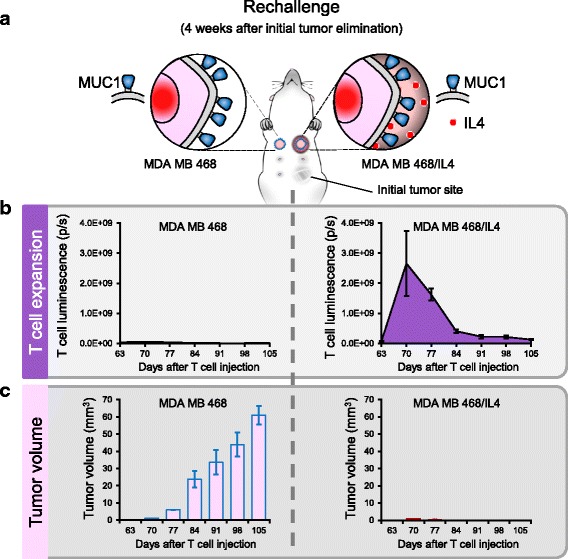
2G.4/7ICR T cells persist long term and retain their anti-tumor activity and tumor selectivity. **a** Schema of tumor rechallenge model. **b** T cell expansion over time as assessed by quantitative bioluminescence imaging at the site of MDA MB 468 (left) and MDA MB 468/IL4 (right) tumor cell injection. **c** Tumor volume as measured by calipers

To assess in vivo persistence and evaluate the tumor selectivity of our 2G.4/7ICR T cells, we rechallenged animals who had initially cleared their IL4-producing tumors (Fig. 4) with 5 × 10^6^ MDA MB 468 cells (right superior mammary fat pad) or 5 × 10^6^ IL4-producing MDA MB 468 cells (MDA MB 468/IL4) on the left superior mammary fat pad (Fig. 5a). As shown in Fig. 5b, tumor rechallenge selectively induced 2G.4/7ICR T cell re-expansion only at the site engrafted with IL4-producing tumor, leading to tumor rejection on that side but contralateral tumor outgrowth (Fig. 5c). These data further illustrate the persistence, proliferative capacity, potency, and cytokine dependency of 2G.4/7ICR T cells.

## Discussion

In the current study, we improved the potency of breast cancer-specific T cells by co-expressing an inverted cytokine receptor (ICR) on CAR T cells targeting MUC1. This combination of modifications: (i) protected transgenic cells from the inhibitory effects of IL4, (ii) enhanced T cell expansion at the tumor site, and (iii) improved the in vitro and in vivo anti-tumor activity of transgenic cells. Importantly, the 4/7ICR did not alter the antigen specificity of the CAR and withdrawal of either antigen or cytokine resulted in rapid T cell contraction, confirming the safety of this strategy for clinical translation.

In nature, T cells require the presence of 3 signals [antigen recognition (signal 1), co-stimulation (signal 2), and cytokine (signal 3)] for potent activation and long-term memory formation, while the absence of any one of these signals substantially impairs T cell function [40, 41]. Indeed, this feature has been exploited clinically in recipients of allogeneic stem cell transplants where alloreactive (GvHD-inducing) T cell activity was blunted by blocking the co-stimulatory CD28 signal [42]. In the current study, we saw similar dysfunction in our transgenic T cells modified to express a first-generation CAR and the 4/7ICR, which provided T cells with signals 1 and 3, respectively. In absence of co-stimulation, our cells displayed an exhausted phenotype, characterized by the upregulation of PD-1/TIM3 and diminished cytolytic function. T cells that received just signals 1 + 2 (2G CAR.MUC1) were similarly dysfunctional and unable to produce tumor control. However, by engineering T cells to receive all 3 signals [T cell activation (signal 1) and co-stimulation (signal 2) - provided by the 2G CAR.MUC1 and cytokine support (signal 3) – provided by the 4/7ICR], we were able to achieve sustained T cell responses, highlighting the importance of recapitulating physiologic T cell signaling in a transgenic cell in order to produce durable anti-tumor effects.

To develop an immunotherapeutic approach for breast cancer, we chose to target an aberrantly glycosylated form of MUC1, which represents a cancer-expressed neoantigen that can be selectively targeted by antibodies and CARs, thereby alleviating concerns associated with “on target off tumor” toxicities [43]. MUC1 was first validated as a transgenic T cell target by Wilkie and colleagues who developed a CAR targeting epitopes in the variable number tandem repeat (VNTR) region that were unmasked due to underglycosylation [32, 44, 45]. Subsequently, June et al. generated a CAR targeting a tumor-specific glycoform (MUC1-Tn), which selectively targeted a range of MUC1+ tumors (leukemia, pancreatic cancer and breast cancer) leaving normal cells including cardiomyocytes, osteoblasts, renal epithelial cells and pulmonary artery endothelial cells untouched [46].

To next ensure that our MUC1-targeted T cells retained effector function in the suppressive tumor microenvironment, we paired our CAR with a chimeric receptor designed to harness and invert the inhibitory effects of tumor-produced IL4. In breast cancer, IL4 is a dominant component of the tumor microenvironment produced both by malignant cells and surrounding adipose tissue [17, 47]. This prototypic Th2 cytokine directly induces the upregulation of anti-apoptotic molecules in malignant cells but also suppresses the effector function of Th1-polarized T cells (Fig. 1 and refs. [14–21]). Hence, we hypothesized that transgenic expression of the 4/7ICR would serve not just to protect our CAR T cells from the inhibitory effects of IL4 (due to the ICR exodomain), but additionally promote their expansion at the tumor site. This proliferative signal is provided to our transgenic cells, courtesy of the IL7 receptor endodomain, which we chose to include given the importance of IL7 signaling in homeostatic proliferation and the maintenance of T cell memory [48–50]. Hence, upon IL4 engagement, our 4/7ICR delivers a prototypic Th1 cytokine signal (signal 3) that supports cell proliferation, persistence and potent anti-tumor effects, as was confirmed in our primary and rechallenge in vivo tumor models.

While co-expressing the 4/7ICR with the CAR improved expansion and anti-tumor activity of T cells in the presence of IL4, this receptor complementation approach of tumor targeting does not address the risk of immune escape due to mutation or loss of the target molecules. Indeed, in patients treated with CD19-targeted CARs, the emergence of CD19-negative relapsed disease is an emerging clinical issue [51–54]. However, given the role of MUC1 and IL4 in tumor progression and metastasis [26–28, 55–58], it is highly unlikely that the tumor will downregulate either one or both of these molecules. Nevertheless, to prevent such eventuality, one could consider combining the 4/7ICR with multiple tumor-targeted CARs.

## Conclusion

In this study, we have demonstrated the feasibility of selectively targeting breast cancer using transgenic T cells equipped to thrive in the suppressive tumor milieu. Our results emphasize the importance of all three signals necessary to fully activate T cells – antigen, co-stimulation, and cytokine for robust and sustained CAR T cell function. The expansion, persistence, potent anti-tumor activity, and safety profile exhibited by the second generation CAR.MUC1 and 4/7ICR modified T cells (2G.4/7ICR) support the clinical translation of this approach for the treatment of patients with breast cancer.

## Additional file

Additional file 1: **Figure S1.** Generation of 2nd generation CAR.MUC1 T cells. (A) Schematic of 2nd generation CAR.MUC1 (2G) retroviral construct. (B) Co-expression of 4/7ICR and 2G CAR as detected by mOrange and anti-IgG, respectively. Summary data (right panel) shows percentage of double-positive cells (mean ± SEM, *n* = 4). (PPTX 298 kb)

## Abbreviations

1G: 1st generation CAR
ALL: Acute lymphoblastic leukemia
B-CLL: B lymphocyte – Chronic lymphocytic leukemia
CARs: Chimeric antigen receptors
Cr: Chromium
FBS: Fetal bovine serum
FFLuc: Firefly luciferase
GFP: Green fluorescent protein
GvHD: Graft versus host disease
i.v.: Intravenous
ICR: Inverted cytokine receptor
IgG2: Immunoglobulin G2
IL4: Interleukin 4
IL7: Interleukin 7
MDSCs: Myeloid-derived suppressor cells
MUC1: Mucin1
PBMCs: Peripheral blood mononuclear cells
PD-1: Programmed cell death protein 1
s.c.: Subcutaneous
scFv: Single-chain variable fragment
TAA: Tumor associated antigen
Th1: Type 1 helper
Th2: Type 2 helper
TIM3: T-cell immunoglobulin and mucin-domain containing-3
TME: Tumor microenvironment
VNTR: Variable number tandem repeat
