# Supplementary material for: CAR T cell therapy for breast cancer: harnessing the tumor milieu to drive T cell activation
Source: J Immunother Cancer. 2018 May 10;6:34. doi: 10.1186/s40425-018-0347-5 (PMC5944113; doi:10.1186/s40425-018-0347-5)
Supplement: Supplementary file 1 — Figure S1. Generation of 2nd generation CAR.MUC1 T cells. (A) Schematic of 2nd generation CAR.MUC1 (2G) retroviral construct. (B) Co-expression of 4/7ICR and 2G CAR as detected by mOrange and anti-IgG, respectively. Summary data (right panel) shows percentage of double-positive cells (mean ± SEM, n = 4). (PPTX 298 kb) [file 40425_2018_347_MOESM1_ESM.pptx]

## Slide 1
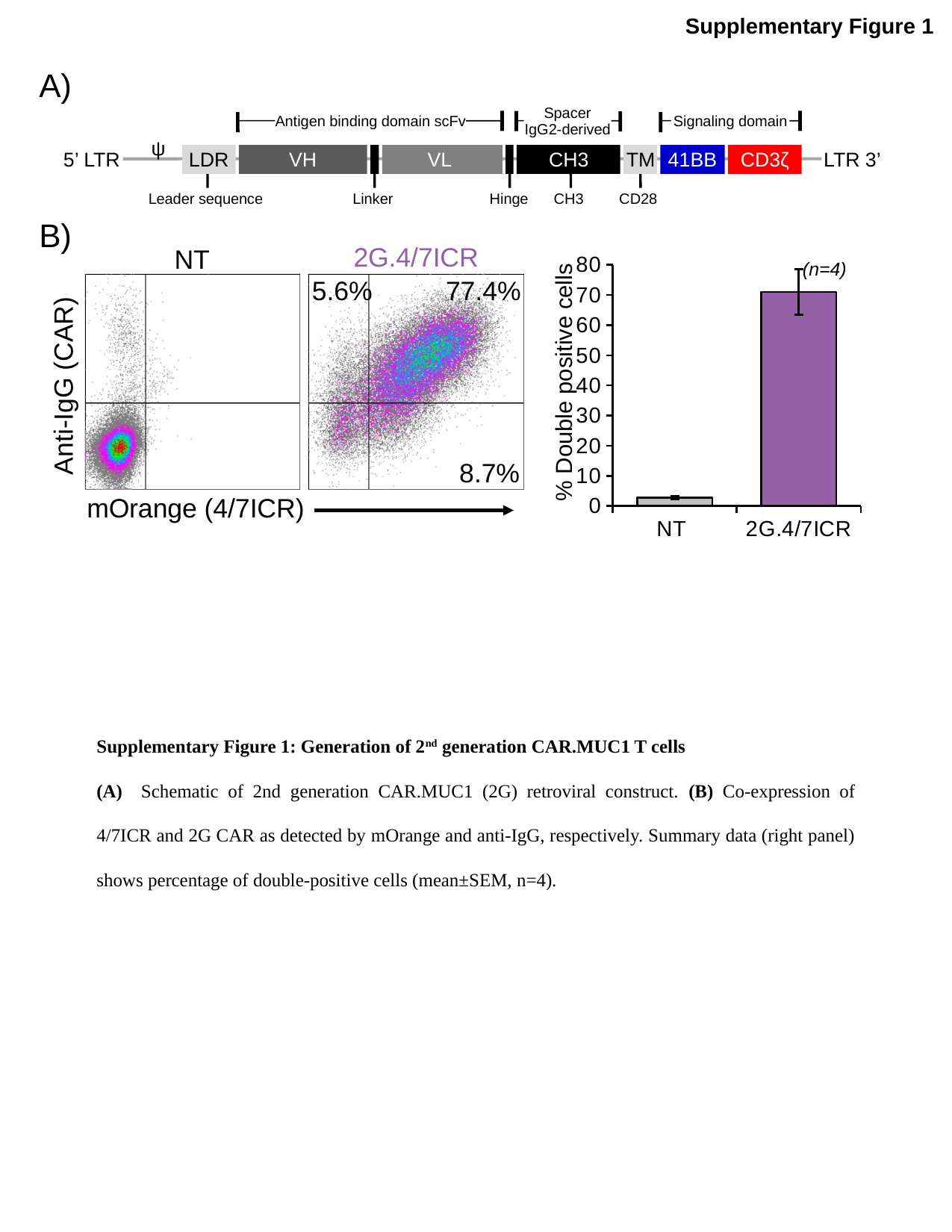

Supplementary Figure 1
A)
Spacer
IgG2-derived
Antigen binding domain scFv
Signaling domain
ψ
5’ LTR
LTR 3’
LDR
VH
VL
CH3
TM
41BB
CD3ζ
Leader sequence
Linker
Hinge
CH3
CD28
B)
2G.4/7ICR
NT
### Chart
| Category | |
|---|---|
| NT | 2.785 |
| 2G.4/7ICR | 71.01 |(n=4)
5.6%
77.4%
% Double positive cells
Anti-IgG (CAR)
8.7%
mOrange (4/7ICR)
Supplementary Figure 1: Generation of 2nd generation CAR.MUC1 T cells
(A) Schematic of 2nd generation CAR.MUC1 (2G) retroviral construct. (B) Co-expression of 4/7ICR and 2G CAR as detected by mOrange and anti-IgG, respectively. Summary data (right panel) shows percentage of double-positive cells (mean±SEM, n=4).
